# *Streptococcus suis* serotype 4: a population with the potential pathogenicity in humans and pigs

**DOI:** 10.1080/22221751.2024.2352435

**Published:** 2024-05-04

**Authors:** Jinlu Zhu, Jianping Wang, Weiming Kang, Xiyan Zhang, Anusak Kerdsin, Huochun Yao, Han Zheng, Zongfu Wu

**Affiliations:** a MOE Joint International Research Laboratory of Animal Health and Food Safety, College of Veterinary Medicine, Nanjing Agricultural University, Nanjing, People’s Republic of China; b Key Lab of Animal Bacteriology, Ministry of Agriculture, Nanjing, People’s Republic of China; c WOAH Reference Lab for Swine Streptococcosis, Nanjing, People’s Republic of China; d Guangdong Provincial Key Laboratory of Research on the Technology of Pig-breeding and Pig-disease Prevention, Guangzhou, People’s Republic of China; e National Institute for Communicable Disease Control and Prevention, Chinese Center for Disease Control and Prevention, Beijing, People’s Republic of China; f Department of Biochemistry and Molecular Biology, Shanxi Key Laboratory of Birth Defect and Cell Regeneration, MOE Key Laboratory of Coal Environmental Pathogenicity and Prevention, Shanxi Medical University, Taiyuan, People’s Republic of China; g Faculty of Public Health, Kasetsart University Chalermphrakiat Sakon Nakhon Province Campus, Sakon Nakhon, Thailand

**Keywords:** *Streptococcus suis* serotype 4, population structure, pathogenicity, antimicrobial susceptibility, prophage, integrative and conjugative elements

## Abstract

*Streptococcus suis* is a major bacterial pathogen in pigs and an emerging zoonotic pathogen. Different *S. suis* serotypes exhibit diverse characteristics in population structure and pathogenicity. Surveillance data highlight the significance of *S. suis* serotype 4 (SS4) in swine streptococcusis, a pathotype causing human infections. However, except for a few epidemiologic studies, the information on SS4 remains limited. In this study, we investigated the population structure, pathogenicity, and antimicrobial characteristics of SS4 based on 126 isolates, including one from a patient with septicemia. We discovered significant diversities within this population, clustering into six minimum core genome (MCG) groups (1, 2, 3, 4, 7-2, and 7-3) and five lineages. Two main clonal complexes (CCs), CC17 and CC94, belong to MCG groups 1 and 3, respectively. Numerous important putative virulence-associated genes are present in these two MCG groups, and 35.00% (7/20) of pig isolates from CC17, CC94, and CC839 (also belonging to MCG group 3) were highly virulent (mortality rate ≥ 80%) in zebrafish and mice, similar to the human isolate ID36054. Cytotoxicity assays showed that the human and pig isolates of SS4 strains exhibit significant cytotoxicity to human cells. Antimicrobial susceptibility testing showed that 95.83% of strains isolated from our labs were classified as multidrug-resistant. Prophages were identified as the primary vehicle for antibiotic resistance genes. Our study demonstrates the public health threat posed by SS4, expanding the understanding of SS4 population structure and pathogenicity characteristics and providing valuable information for its surveillance and prevention.

## Introduction

*Streptococcus suis* is an important pathogen in the pig industry, causing septicemia, meningitis, and sudden death in pigs, imposing substantial economic losses on the industry [1]. Moreover, *S. suis* is an emerging zoonotic pathogen that can be transmitted to humans by contact with diseased animals or contaminated raw pork products [2,3]. Indeed, human cases of *S. suis* have been reported worldwide. Particularly in Vietnam and Thailand, *S. suis* was responsible for thousands of human disease cases and has been identified as one of the most prevalent causes of adult bacterial meningitis [2,4,5]. Based on the capsular polysaccharide (CPS) antigenicity variation, *S. suis* can be classified into 29 serotypes (1–19, 21, 23–25, 27–31, 1/2) [6,7]. Also, serotype Chz and 27 novel *cps* loci (NCLs) have been identified from non-typeable isolates based on differences in the *cps* gene cluster [8–13]. To date, 11 serotypes have been documented as capable of inducing human infections, consisting of serotypes 1, 2, 4, 5, 7, 9, 14, 16, 21, 24, and 31 [14,15].

Although the distribution of serotypes in clinical cases may vary across geographic locations, *S. suis* serotype 2 is universally recognized as the most prevalent pathotype in both swine and humans worldwide [1]. Therefore, most studies have focused on *S. suis* serotype 2. However, different serotypes exhibit diverse characteristics in population structure and pathogenicity. In recent years, we have noticed an increase in the isolation rate of *S. suis* serotype 4 in pig populations. In addition, the report of the first human case caused by serotype 4 dated back to 1988 in the Netherlands, and the second human case caused by ST94 strain of serotype 4 was reported in 2018 in Thailand [16,17]. Notably, *S. suis* serotype 4 has been identified as one of the most prevalent serotypes in diseased pigs in Asia, and strains of this serotype have also been detected in healthy and diseased pigs in specific European and North American nations [6,18–21]. In a recent study, Murray *et al.* identified 10 pathogenic lineages of *S. suis* based on isolates sampled from healthy and diseased pigs, wild boar, and humans, from Asia, North America, Europe, and Australia [22]. They revealed that 80.36% of *S. suis* serotype 4 isolates (90/112) belong to these pathogenic lineages [22]. These findings highlight the significance of *S. suis* serotype 4 in swine streptococcusis. However, except for the information in human case reports and a few epidemiologic studies, the information on *S. suis* serotype 4 remains exceedingly limited.

In this study, a total of 126 *S. suis* serotype 4 genomes from eight different countries were analyzed, including one isolate from a patient with septicemia. A systematic bioinformatic analysis was conducted to investigate their population structure, phylogenetic relationship, putative virulence-associated genes, antibiotic resistance genes, and dissemination vehicles of antibiotic resistance genes. Additionally, animal infection experiments, human cell cytotoxicity assays, and antimicrobial susceptibility testing were performed on *S. suis* serotype 4 strains isolated from pigs and human to assess their pathogenicity and resistance characteristics. This study contributes to our understanding of *S. suis* serotype 4 and provides valuable information for the surveillance and prevention of this serotype.

## Materials and methods

### Bacterial strains and culture conditions

To investigate the characteristics of *S. suis* serotype 4 population, 48 strains isolated from our labs and 78 genomes from NCBI database were used in this study, as shown in Table 1. The 48 strains were collected from diseased and healthy pigs in China between 2014 and 2022. All strains were confirmed to be *S. suis* by analyzing their 16S rRNA gene sequences [23] and *recN* gene [24]. Furthermore, these strains were identified as serotype 4 by the PCR method based on the serotype 4 specific *wzy* gene [25] and the agglutination test using serotype 4 specific serum purchased from Statens Serum Institute (Copenhagen, Denmark). In addition, 78 *S. suis* serotype 4 genomes downloaded from the NCBI database originated from eight different countries, consisting of 30 from UK, 21 from China, ten from Canada, seven from Thailand, four from the Netherlands, four from the USA, and one each from Denmark and Spain. These genomes harboured *S. suis* serotype 4 specific *wzy* gene and were isolated from 1991 to 2019. The human strain ID36054 was isolated from a patient with septicemia reported in 2018 [17,26]. Strains were cultured in Todd-Hewitt broth (THB, Hope Bio-Technology Co., Ltd, China) and plated on THB agar containing 5% sheep blood at 37°C and 5% CO_2_.

**Table 1.** The information of S. suis serotype 4 strains used in this study.

Lineages	Strains	Accession number	ST	CC	MCG	Country	Date	Host	Isolation	Source
Lineage 1	CPD27	SAMN12784764	17	17	1	China	2010	Pig	/	NCBI
	LSSP193	SAMN26554949	17	17	1	China	2017	Pig	/	NCBI
	SC5B93	SAMN14687788	17	17	1	China	2019	Pig	/	NCBI
	ND7	SAMN34237859	17	17	1	China	2014	Diseased pig	Lung	This study
	ND83	SAMN34237860	17	17	1	China	2014	Diseased pig	Lung	This study
	ND84	SAMN34237861	17	17	1	China	2014	Diseased pig	Lung	This study
	ND90	SAMN34237862	17	17	1	China	2014	Diseased pig	Lung	This study
	WUSS270	SAMN34237819	17	17	1	China	2017	Healthy pig	Tonsil	This study
	WUSS304	SAMN34237824	17	17	1	China	2017	Healthy pig	Tonsil	This study
	WUSS388	SAMN34237830	17	17	1	China	2017	Healthy pig	Tonsil	This study
	WUSS406	SAMN34237833	17	17	1	China	2017	Healthy pig	Tonsil	This study
	2018WUSS011	SAMN34237836	17	17	1	China	2018	Healthy pig	Tonsil	This study
	CPD30	SAMN12784768	850	17	1	China	2011	Pig	/	NCBI
	WUSS303	SAMN34237823	850	17	1	China	2017	Healthy pig	Tonsil	This study
	2021WUSS074	SAMN34237846	2224	17	1	China	2021	Healthy pig	Tonsil	This study
	2021WUSS078	SAMN34237850	2224	17	1	China	2021	Healthy pig	Tonsil	This study
	2021WUSS076	SAMN34237848	2224	17	1	China	2021	Healthy pig	Tonsil	This study
	2021WUSS079	SAMN34237851	2224	17	1	China	2021	Healthy pig	Tonsil	This study
	CPD39	SAMN12784777	2235	17	1	China	2010	Pig	/	NCBI
	CPD29	SAMN12784766	2235	17	1	China	2010	Pig	/	NCBI
	LSSP132	SAMN26111036	2236	17	1	China	2017	Pig	Brain	NCBI
	GD-0057	SAMEA3595235	17	17	1	Netherlands	2004	Diseased pig	CSF	NCBI
	GD-0073	SAMEA3595242	17	17	1	Netherlands	2005	Diseased pig	CSF	NCBI
	GD-0098	SAMEA3595254	17	17	1	Netherlands	2006	Diseased pig	CSF	NCBI
	MA6	SAMN21439331	17	17	1	Netherlands	2017	Diseased pig	Brain/blood	NCBI
	MA2	SAMN21439327	17	17	1	Canada	2016	Pig	Tonsil	NCBI
	DB1V3-4A	SAMN14932575	17	17	1	Canada	2016	Pig	/	NCBI
	40439	SAMN13975665	17	17	1	USA	2017	Pig	/	NCBI
	40458	SAMN13975647	17	17	1	USA	2016	Pig	/	NCBI
	JT9	SAMN29093732	17	17	1	Spain	2018	Pig	Brain	NCBI
Lineage 2	SS967	SAMN14933159	23	87	2	UK	2010	Pig	/	NCBI
	SS1048	SAMN14932752	23	87	2	UK	2010	Pig	/	NCBI
	LSS20	SAMN14932632	23	87	2	UK	2010	Pig	/	NCBI
	S14O	SAMEA3234013	23	87	2	UK	2010	Diseased pig	Lung	NCBI
	S97A	SAMEA3234092	23	87	2	UK	2010	Pig	Lung	NCBI
	LS0M	SAMEA3233887	23	87	2	UK	2010	Healthy pig	Tonsil	NCBI
	SS967_2	SAMEA1316593	23	87	2	UK	2012	Pig	/	NCBI
	SS1048_2	SAMEA1316599	23	87	2	UK	2012	Pig	/	NCBI
	LSS20_2	SAMEA1316631	23	87	2	UK	2012	Pig	/	NCBI
	TMW-SS042	SAMN14933224	23	87	2	UK	2013	Pig	/	NCBI
	LSS94	SAMN14932706	862	87	2	UK	2011	Pig	/	NCBI
	LS4P	SAMEA3233921	862	87	2	UK	2011	Healthy pig	Tonsil	NCBI
	LSS94_2	SAMEA1316533	862	87	2	UK	2012	Pig	/	NCBI
	6407^a^	SAMN02905150	54	54	3	Denmark	/	Pig	/	NCBI
Lineage 3	942	SAMN08295926	94	94	3	China	2013	Pig	Tonsil	NCBI
	944	SAMN08295927	94	94	3	China	2013	Pig	Tonsil	NCBI
	1044	SAMN08295942	94	94	3	China	2013	Pig	Tonsil	NCBI
	SH1510	SAMN09460428	94	94	3	China	2015	Pig	Lung	NCBI
	LSSP204	SAMN26554953	94	94	3	China	2017	Pig	/	NCBI
	LSSP213	SAMN26554957	94	94	3	China	2018	Pig	/	NCBI
	LSSP237	SAMN28125328	94	94	3	China	2018	Pig	/	NCBI
	ND6	SAMN34237858	94	94	3	China	2014	Diseased pig	Lung	This study
	WUSS026	SAMN34237817	94	94	3	China	2017	Healthy pig	Tonsil	This study
	WUSS273	SAMN34237820	94	94	3	China	2017	Healthy pig	Tonsil	This study
	2021WUSS075	SAMN34237847	94	94	3	China	2021	Healthy pig	Tonsil	This study
	2021WUSS077	SAMN34237849	94	94	3	China	2021	Healthy pig	Tonsil	This study
	2021WUSS080	SAMN34237852	94	94	3	China	2021	Healthy pig	Tonsil	This study
	WUSS326	SAMN34237826	1175	94	3	China	2017	Healthy pig	Tonsil	This study
	WUSS329	SAMN34237827	1175	94	3	China	2017	Healthy pig	Tonsil	This study
	2018WUSS156	SAMN34237840	2220	94	3	China	2018	Diseased pig	/	This study
	SS1042	SAMN14932750	911	94	3	UK	2010	Pig	/	NCBI
	SS1041	SAMN14932749	911	94	3	UK	2010	Pig	/	NCBI
	SS1040	SAMN14932748	911	94	3	UK	2010	Pig	/	NCBI
	S14J	SAMEA3234009	911	94	3	UK	2010	Diseased pig	Lung	NCBI
	S14K	SAMEA3234010	911	94	3	UK	2010	Diseased pig	Lung	NCBI
	S14L	SAMEA3234011	911	94	3	UK	2010	Diseased pig	Lung	NCBI
	SS1042_2	SAMEA1316637	911	94	3	UK	2012	Pig	/	NCBI
	SS1041_2	SAMEA1316538	911	94	3	UK	2012	Pig	/	NCBI
	SS1040_2	SAMEA1316654	911	94	3	UK	2012	Pig	/	NCBI
	TMW-SS070	SAMN14933250	911	94	3	UK	2014	Pig	/	NCBI
	TRG6	SAMN31277175	94	94	3	Thailand	2010	Diseased pig	Lung	NCBI
	ID36054	SAMN31277174	94	94	3	Thailand	2011	Homo sapiens	Blood	NCBI
	ID34693	SAMN31277178	94	94	3	Thailand	2011	Pig	Tonsil	NCBI
	ID34704	SAMN31277179	94	94	3	Thailand	2011	Pig	Tonsil	NCBI
	ID34545	SAMN31277176	1689	94	3	Thailand	2011	Pig	Tonsil	NCBI
	ID34553	SAMN31277177	1689	94	3	Thailand	2011	Pig	Tonsil	NCBI
	ID34572	SAMN31277173	1689	94	3	Thailand	2011	Healthy pig	Tonsil	NCBI
	1602956	SAMN14932473	94	94	3	Canada	2014	Pig	/	NCBI
	1665814	SAMN14932502	1175	94	3	Canada	2014	Pig	/	NCBI
	CPD36	SAMN12784774	485	839	3	China	2013	Pig	/	NCBI
	LSSP145	SAMN26111047	485	839	3	China	2017	Pig	Lung	NCBI
	LSSP144	SAMN26111046	485	839	3	China	2017	Pig	Lung	NCBI
	WUSS346	SAMN34237829	485	839	3	China	2017	Healthy pig	Tonsil	This study
	2018WUSS160	SAMN34237841	485	839	3	China	2018	Diseased pig	Spleen	This study
	MY1C3-3C	SAMN14932601	839	839	3	Canada	2016	Pig	/	NCBI
	MA4T3-4C	SAMN14932592	839	839	3	Canada	2016	Pig	/	NCBI
	40436	SAMN13975668	839	839	3	USA	2016	Pig	/	NCBI
	D16-010378	SAMN14932573	977	108	3	Canada	/	Pig	/	NCBI
	1652716	SAMN14932497	977	108	3	Canada	2014	Pig	/	NCBI
	1607743	SAMN14932477	977	108	3	Canada	2014	Pig	/	NCBI
	WUSS390	SAMN34237831	977	108	3	China	2017	Healthy pig	Tonsil	This study
Lineage 4	LOLA-SS006	SAMN14932618	856	28	4	UK	2010	Pig	/	NCBI
	LL-U	SAMEA3233876	856	28	4	UK	2010	Diseased pig	Lung	NCBI
	91-178-2215	SAMN14932532	2233	1372	4	Canada	1991	Pig	/	NCBI
Lineage 5	WUSS435	SAMN34237834	1067	/	7-2	China	2017	Diseased pig	/	This study
	WUSS436	SAMN34237835	1067	/	7-2	China	2017	Healthy pig	Tonsil	This study
	2019WUSS015	SAMN34237842	2221	/	7-2	China	2019	Healthy pig	Tonsil	This study
	2019WUSS016	SAMN34237843	2221	/	7-2	China	2019	Healthy pig	Tonsil	This study
	2020WUSS060	SAMN34237845	2223	/	7-2	China	2020	Healthy pig	Tonsil	This study
	2022WUSS016	SAMN34237853	2225	/	7-2	China	2021	Healthy pig	Tonsil	This study
	2022WUSS017	SAMN34237854	2226	/	7-2	China	2021	Healthy pig	Tonsil	This study
	WUSS228	SAMN34237818	2237	/	7-2	China	2017	Healthy pig	Tonsil	This study
	WUSS309	SAMN34237825	2237	/	7-2	China	2017	Healthy pig	Tonsil	This study
	WUSS285	SAMN34237821	2238	/	7-2	China	2017	Healthy pig	Tonsil	This study
	WUSS299	SAMN34237822	2238	/	7-2	China	2017	Healthy pig	Tonsil	This study
	WUSS333	SAMN34237828	2238	/	7-2	China	2017	Healthy pig	Tonsil	This study
	WUSS399	SAMN34237832	2239	/	7-2	China	2017	Healthy pig	Tonsil	This study
	CPD3	SAMN12784778	935	/	7-3	China	2014	Pig	/	NCBI
	HA1003	SAMN09460429	1006	/	7-3	China	2010	Healthy pig	Tonsil	NCBI
	1367	SAMN08295994	2229	/	7-3	China	2013	Pig	Tonsil	NCBI
	1369	SAMN08295995	2229	/	7-3	China	2013	Pig	Tonsil	NCBI
	2018WUSS056	SAMN34237837	2218	/	7-3	China	2018	Healthy pig	Tonsil	This study
	2018WUSS108	SAMN34237838	2219	/	7-3	China	2018	Healthy pig	Tonsil	This study
	2018WUSS109	SAMN34237839	2219	/	7-3	China	2018	Healthy pig	Tonsil	This study
	2020WUSS059	SAMN34237844	2222	/	7-3	China	2020	Healthy pig	Tonsil	This study
	2022WUSS018	SAMN31099739	2057	/	7-3	China	2021	Healthy pig	Tonsil	This study
	2022WUSS019	SAMN34237855	2227	/	7-3	China	2021	Healthy pig	Tonsil	This study
	2022WUSS020	SAMN34237856	2057	/	7-3	China	2021	Healthy pig	Tonsil	This study
	2022WUSS056	SAMN34237857	2228	/	7-3	China	2022	Healthy pig	Tonsil	This study
	2022WUSS141	SAMN31099758	2066	/	7-3	China	2022	Healthy pig	Tonsil	This study
	LSS33	SAMN14932646	895	/	7-3	UK	2011	Pig	/	NCBI
	LS3A	SAMEA3233909	895	/	7-3	UK	2011	Pig	/	NCBI
	SS1028	SAMEA1316687	908	/	7-3	UK	2012	Pig	/	NCBI
	S12X	SAMEA3233997	908	/	7-3	UK	2010	Diseased pig	Brain	NCBI
	270-6A	SAMN14933322	2234	/	7-3	UK	2013	Pig	/	NCBI
	32052	SAMN13975691	1209	/	7-3	USA	2015	Pig	/	NCBI

/: unassigned.

^a^ Except for strain 6407, the remaining genomes from MCG group 3 were assigned to lineage 3.

### Bioinformatic analysis of *S. suis* serotype 4 genomes

The draft genomes of 48 strains isolated from our labs were sequenced using Illumina NovaSeq PE150 at Beijing Novogene Bioinformatics Technology Co., Ltd (China). The multilocus sequence type (MLST) and the minimum core genome (MCG) groups of 126 *S. suis* serotype 4 genomes were determined using the PubMLST database [27] and a previously established approach [28], respectively. Additionally, the global optimal eBURST (goeBURST) analysis [29] was employed to classify clonal complexes (CCs). Bowtie 2 was used to identify single-nucleotide polymorphisms (SNPs) within *S. suis* serotype 4 genomes, using the genome sequence of SC84 (accession number FM252031) as a reference. The mutational SNP sites were selected based on the procedure described in a previous study [28], and the phylogenetic tree was constructed using the maximum likelihood method by FastTree v2.1.10. As an outgroup, *Streptococcus pneumoniae* ATCC 700669 (NC_011900) was used to root the tree, and tree visualization was completed using tvBOT v2.5.2 [30]. The distribution of 35 putative virulence-associated genes of *S. suis*, as described in previous reports [31,32] (listed in Table S1), was investigated among the 126 *S. suis* serotype 4 genomes. Genes with coverage <80% or a nucleotide sequence identity <80% were determined to be absent. Antibiotic resistance gene analysis was performed using ResFinder 4.1 [33]. The whole genome sequencing data was input for ResFinder 4.1, with parameters set at ≥ 80% identity over ≥ 80% coverage of the reference gene. Prophages were predicted using PHASTER [34]. The prophage is incomplete if the score is less than 60, questionable if it is between 70 and 90, and intact if it is greater than 90 [34]. The integrative and conjugative elements (ICEs) analysis based on the major insertion hotspots *rplL* and *rum* loci was conducted according to a previous study [35]. The conjugative plasmids carrying antibiotic resistance genes were predicted using VRprofile2 [36] and oriTfinder [37]. The obtained mobile genetic element (MGE) sequences were further annotated using an online RAST server [38], followed by comparison and visualization using the BLASTn programme inserted within Easyfig 2.2.3 software [39].

### Animal infection experiments

Animal infection experiments were conducted at the Laboratory Animal Center of Nanjing Agricultural University (Permit number: SYXK (Su) 2021-0086). According to the population structure of *S. suis* serotype 4, we selected 34 of 48 strains isolated from our labs, along with the human isolate ID36054, for the zebrafish infection experiment. The infection protocol for zebrafish was described in our previous studies [40,41]. Briefly, bacteria were collected during the mid-log phase, washed twice in PBS, and then adjusted to the proper infection concentrations in PBS. Each experimental group comprised 15 zebrafish, with each fish receiving an intraperitoneal injection containing 3 × 10^6^ CFU of *S. suis* in 20 µL of PBS. The mortality was recorded from 12 h until 96 h after the challenge. Then, strains highly pathogenic to zebrafish (mortality rate ≥ 80%) were chosen for the mouse infection experiment. Six-week-old BALB/c mice were purchased from the Shanghai SLAC Laboratory Animal Co., Ltd (China). Each experimental group comprised 10 mice, and they were intraperitoneally injected with 3 × 10^8^ CFU of *S. suis* per mouse. After infection, the mortality of the mice was monitored for 10 days. In the animal infection experiments, the highly virulent *S. suis* serotype 2 strain SC070731 [42], non-virulent *S. suis* serotype 9 strain SH040917 [43], and an equivalent volume of PBS were included as controls. According to our previous reports [14,40,41], a strain with a mortality rate ≥ 80% in zebrafish and mice was defined as highly virulent. The Log-rank (Mantel–Cox) test was used to compare the survival curves of zebrafish and mice infected with *S. suis* strains.

### Human cell cytotoxicity assays

Human lung adenocarcinoma cells (A549) and human brain microvascular endothelial cells (hBMEC) were used for cell cytotoxicity assays, according to previous reports [26,44–46]. The human isolate ID36054 and four pig isolates 2021WUSS075, 2018WUSS156, ND90, and 2018WUSS160, with a mortality rate of ≥ 80% in zebrafish and mice, were selected for cell cytotoxicity assays. Strains were cultured and collected during the mid-log phase and were prepared at a concentration of 1 × 10^7^ CFU/mL in THB medium. The A549 and hBMEC cell lines were purchased from the Cell Resource Center, IBMS, CAMS/PUMC (Beijing, China) and cultured in F12/DMEM (Gibco, Carlsbad, USA) and DMEM (Hyclone, Beijing, China), respectively, each supplemented with 10% fetal bovine serum, and maintained at 37°C in an atmosphere of 5% CO_2_. For each experiment, A549 or hBMEC cells were plated into 24-well flat-bottom plates at 3 × 10^5^ cells/well in 1 mL corresponding medium and maintained in 5% CO_2_ at 37°C for 48 h to allow cell confluence. Then, the cell number reached approximately 1 × 10^6^ cells/well before the infection assays. The medium was changed every 24 h. In the cytotoxicity assay, *S. suis* (1 × 10^7^ CFU/well) were added to A549 or hBMEC cells at the multiplicity of infection (MOI) of 10, incubated for 3 and 6 h at 37°C with 5% CO_2_, respectively. Following the manufacturer's instructions, the supernatant was collected to measure lactate dehydrogenase (LDH) release using the LDH Cytotoxicity Assay-Fluorescence Kit (Thermo Fisher). The unpaired *t*-test was used to compare the percentage of cytotoxicity of *S. suis* serotype 4 strains. This experiment has been performed with a minimum of three independent biological replicates.

### Antimicrobial susceptibility testing

Minimum inhibitory concentrations (MICs) were determined using the broth microdilution method following the guidelines outlined in the Clinical and Laboratory Standards Institute (CLSI) document (M31-A3). The following 23 antibiotics of 11 categories were tested: *β*-lactam antibiotics (penicillin, cefotaxime, and amoxicillin), rifamycin (rifampin), glycopeptide (vancomycin), quinolones (marbofloxacin and enrofloxacin), oxazolidinone (linezolid), amphenicols (chloramphenicol and florfenicol), macrolides (tilmicosin, azithromycin, and erythromycin), pleuromutilins (valnemulin and tiamulin), aminoglycosides (gentamicin, streptomycin, kanamycin, and spectinomycin), lincosamides (clindamycin and lincomycin), and tetracyclines (doxycycline and tetracycline). These antibiotics were tested at concentrations ranging from 0.5–256 µg/mL, with breakpoints for resistance according to the CLSI document (VET08-ED4) and guidelines provided by the European Committee on Antimicrobial Susceptibility Testing (EUCAST) (https://www.eucast.org/), as listed in our previous study [41].

## Results

### Population structure and phylogenetic relationship of *S. suis* serotype 4

**Figure 1.**
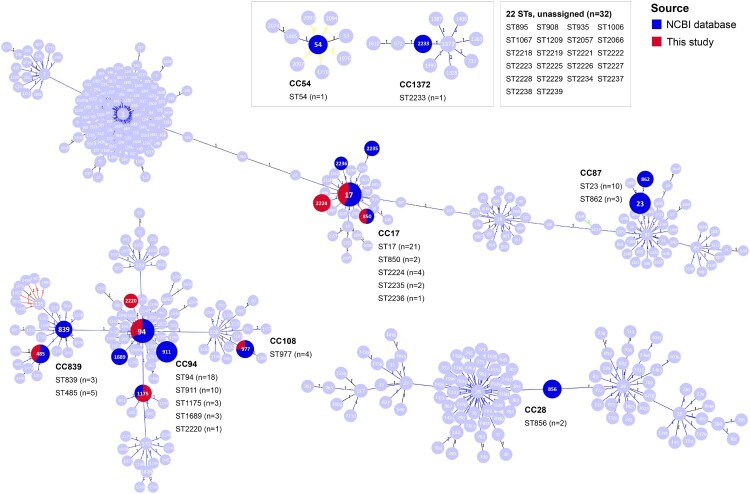
A goeBURST analysis of STs of the *S. suis* serotype 4 population. Numbers in circles indicate partial STs in *S. suis* MLST database. Deep blue circles and red circles indicate STs identified in the *S. suis* serotype 4 genomes downloaded from the NCBI database and sequenced in this study, respectively. “*n*” indicates the number of genomes. STs connected by a line mean that they have six identical alleles. Clusters of linked STs correspond to CCs.

**Figure 2.**
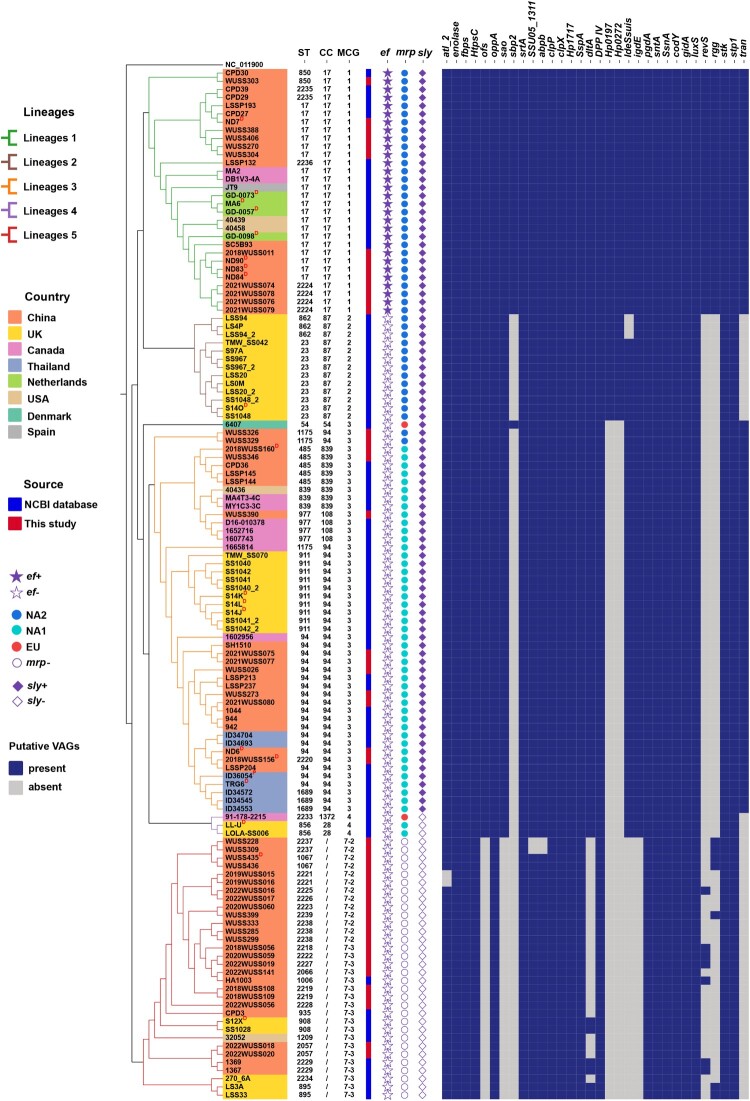
The phylogenetic tree and information of STs, CCs, MCG groups, and putative virulence-associated genes (VAGs) for the *S. suis* serotype 4 population. The superscripts “D” and “P” indicate strains originating from diseased pigs and human patients, respectively. The phylogenetic tree was constructed based on the SNPs of the core genome. The *S. pneumoniae* ATCC 700669 (NC_011900) was used as an outgroup to root the tree.

MLST analysis showed that the *S. suis* serotype 4 population exhibited significant genetic diversity, with 40 distinct sequence types (STs) found throughout 126 genomes (Table 1). Most prevalent were ST17 (*n* = 21) and ST94 (*n* = 18), followed by ST23 (*n* = 10), ST911 (*n* = 10), ST485 (*n* = 5), ST977 (*n* = 4), ST2224 (*n* = 4), ST839 (*n* = 3), ST862 (*n* = 3), ST1175 (*n* = 3), ST1689 (*n* = 3), and ST2238 (*n* = 3). Each of the remaining ST54, ST850, ST856, ST895, ST908, ST935, ST1006, ST1067, ST1209, ST2057, ST2066, ST2218-2223, ST2225-2229, ST2233-2237, and ST2239 consisted of two or one genomes. As shown in Figure 1, 18 STs (94/126 genomes, 74.60%) belonged to eight CCs, consisting of CC94 (ST94, ST911, ST1175, ST1689, and ST2220), CC17 (ST17, ST850, ST2224, ST2235, and ST2236), CC87 (ST23 and ST862), CC839 (ST839 and ST485), CC108 (ST977), CC28 (ST856), CC54 (ST54), and CC1372 (ST2233). In contrast, the remaining 22 STs (32/126 genomes, 25.40%) did not belong to any CCs. As shown in Figure 2, according to MCG analysis, 126 genomes belonged to six MCG groups, including MCG groups 1, 2, 3, 4, 7-2, and 7-3. Notably, two main CCs, CC17 (30/126 genomes, 23.81%) and CC94 (35/126 genomes, 27.78%), belonged to MCG groups 1 and 3, respectively. Most strains isolated from diseased pigs belonged to these two CCs. Moreover, the strain ID36054, responsible for human infections, also belonged to CC94. Based on an evolutionary tree constructed from SNPs in core genomes, the 126 genomes were divided into five lineages (Figure 2). Lineages 1 and 2 comprised MCG groups 1 and 2, respectively. Except for strain 6407, the remaining genomes from MCG group 3 were assigned to lineage 3. Lineage 4 was composed of MCG group 4. MCG groups 7-2 and 7-3 were collectively categorized as lineage 5.

### Distribution of putative virulence-associated genes in *S. suis* serotype 4

Thirty-five putative virulence-associated genes preferentially present in highly pathogenic *S. suis* serotype 2 strains [31,32] were analyzed among *S. suis* serotype 4 genomes. As shown in Figure 2, 18 of 35 putative virulence-associated genes were present in all *S. suis* serotype 4 genomes. The genes *atl_2* (124/126, 98.41%), *SSU05*_*1311* (124/126, 98.41%), and *abpb* (124/126, 98.41%) were present in most *S. suis* serotype 4 genomes. The remaining 14 of 35 putative virulence-associated genes showed distribution correlated with the MCG groups. All genomes within MCG group 1 exhibited the genotype of *ef*+/*mrp*+/*sly*+. For MCG groups 2 and 3, all genomes exhibited the genotype of *ef*-/*mrp*+/*sly*+. For MCG group 4, all genomes exhibited the genotype of *ef*-/*mrp*+/*sly*-. However, all three classical virulence marker genes were absent in MCG groups 7-2 and 7-3. Based on variation in the central region of the gene, *mrp* was classified as EU, NA1, and NA2 subtypes [14]. MCG groups 1 and 2 are all subtype NA2, while MCG groups 3 and 4 are mostly NA1 (Figure 2). Genes *dltA*, *IdeSsuis*, *igdE*, *ofs*, and *sao* were widespread in serotype 4 genomes, except for MCG groups 7-2 and 7-3. In contrast, the *sbp2* gene was primarily distributed in MCG group 1, and *hp0197* and *hp0272* were only present in MCG groups 1 and 2. The regulatory genes *rgg* and *revs* were predominantly distributed in MCG group 1, and the *tran* gene was present in MCG groups 1 and 3. Importantly, it should be noted that all 35 putative virulence-associated genes were found to be present in MCG group 1 (Figure 2).

### The results of animal infection experiments

To assess the virulence of the *S. suis* serotype 4 population, we selected 35 representative strains based on their distribution in the phylogenetic tree and their host for the zebrafish infection experiment. One strain ID36054 originated from a patient, eight strains (ND7, ND83, ND84, ND90, ND6, 2018WUSS156, 2018WUSS160, and WUSS435) originated from diseased pigs, and the remaining from healthy pigs (Table 2). As shown in Table 2, the mortality rate of zebrafish infected with nine strains (ID36054, WUSS406, 2018WUSS011, ND84, ND90, 2021WUSS075, 2018WUSS156, WUSS346, and 2018WUSS160) reached or exceeded 80%, classified as highly virulent strains. The survival curves of zebrafish infected with strains ID36054, 2018WUSS011, ND84, 2021WUSS075, WUSS346, and 2018WUSS160 showed no significant difference compared to zebrafish infected with the highly virulent control strain SC070731. Notably, strains WUSS406, 2018WUSS011, 2021WUSS075, and WUSS346 were isolated from healthy pigs. The abovementioned nine strains belonged to CC17 (4/11 representatives, 36.36%), CC94 (3/8 representatives, 37.50%), and CC839 (2/2 representatives, 100.00%), respectively. They belong to MCG groups 1 and 3, and harbour numerous crucial genes associated with virulence (Figure 2). There are also five strains (WUSS270, WUSS388, ND83, WUSS303, and WUSS026) with mortality rates between 50% and 80%, which also belong to CC17 and CC94. The mortality rate of zebrafish infected with the remaining strains, including all representative strains of MCG groups 7-2 and 7-3 (13/13 representatives, 100.00%), was <50%, classified as lowly virulent strains (Table 2). The gross pathology of zebrafish infected with highly virulent, moderately virulent, lowly virulent, and non-virulent strains of *S. suis* serotype 4 is shown in Figure S1.

**Table 2.** The results of the zebrafish infection experiment.

Strains	ST	CC	Host^a^	Deaths at different post-infection time points								Total deaths	Mortality rate (%)	*P* value	Significance^b^
				12 h	24 h	36 h	48 h	60 h	72 h	84 h	96 h				
WUSS270	17	17	H	0	4	3	1	1	0	0	0	9	60.00	0.0008	***
WUSS304	17	17	H	0	2	3	0	0	0	0	0	5	33.33	<0.0001	****
WUSS388	17	17	H	0	7	4	0	0	0	0	0	11	73.33	0.0051	**
WUSS406	17	17	H	0	9	1	0	0	0	1	1	12	80.00	0.0225	*
2018WUSS011	17	17	H	2	8	4	0	0	0	0	0	14	93.33	0.1408	ns
ND7	17	17	D	0	0	0	0	0	0	1	0	1	6.67	<0.0001	****
ND83	17	17	D	10	0	1	0	0	0	0	0	11	73.33	0.8457	ns
ND84	17	17	D	13	0	0	0	0	0	0	0	13	86.67	0.0921	ns
ND90	17	17	D	0	8	3	0	1	0	0	0	12	80.00	0.0142	*
WUSS303	850	17	H	0	2	5	1	0	0	0	0	8	53.33	0.0001	***
2021WUSS076	2224	17	H	1	1	4	0	0	1	0	0	7	46.67	0.0002	***
ID36054	94	94	*P*	5	2	5	1	1	0	0	0	14	93.33	0.2186	ns
WUSS026	94	94	H	1	3	4	1	1	0	0	0	10	66.67	0.0026	**
WUSS273	94	94	H	0	0	4	0	0	0	0	0	4	26.67	<0.0001	****
2021WUSS075	94	94	H	6	5	1	2	0	0	0	0	14	93.33	0.8088	ns
2021WUSS077	94	94	H	0	2	0	1	0	0	0	0	3	20.00	<0.0001	****
ND6	94	94	D	3	0	1	0	0	0	0	0	4	26.67	0.0007	***
WUSS329	1175	94	H	0	0	3	0	1	0	0	0	4	26.67	<0.0001	****
2018WUSS156	2220	94	D	0	13	0	0	0	0	0	0	13	86.67	0.0405	*
WUSS346	485	839	H	8	2	4	0	0	0	0	0	14	93.33	0.8375	ns
2018WUSS160	485	839	D	0	15	0	0	0	0	0	0	15	100.00	0.0979	ns
WUSS390	977	108	H	0	9	1	0	0	0	0	0	10	66.67	0.0080	**
WUSS285	2238	/	H	2	0	1	0	1	0	0	0	4	26.67	0.0002	***
WUSS309	2237	/	H	2	1	0	0	0	0	0	0	3	20.00	0.0003	***
WUSS399	2239	/	H	0	0	4	0	0	0	0	0	4	26.67	<0.0001	****
WUSS435	1067	/	D	1	0	1	1	0	0	0	0	3	20.00	<0.0001	****
2018WUSS056	2218	/	H	0	0	0	0	0	0	0	0	0	0.00	<0.0001	****
2018WUSS108	2219	/	H	1	2	1	1	1	0	0	0	6	40.00	0.0004	***
2020WUSS059	2222	/	H	0	1	0	1	0	0	0	0	2	13.33	<0.0001	****
2022WUSS016	2225	/	H	4	0	2	0	1	0	0	0	7	46.67	0.0057	**
2022WUSS017	2226	/	H	1	3	1	0	0	0	0	0	5	33.33	0.0004	***
2022WUSS018	2057	/	H	0	0	0	0	0	0	0	0	0	0.00	<0.0001	****
2022WUSS019	2227	/	H	0	1	0	1	0	0	1	0	3	20.00	<0.0001	****
2022WUSS056	2228	/	H	0	0	0	0	0	0	0	0	0	0.00	<0.0001	****
2022WUSS141	2066	/	H	0	1	0	1	0	0	0	0	2	13.33	<0.0001	****
SC070731	7	1	D	5	9	0	0	0	0	0	0	14	93.33	–	–
SH040917	/	/	H	0	0	0	0	0	0	0	0	0	0.00	<0.0001	****
PBS				0	0	0	0	0	0	0	0	0	0.00	<0.0001	****

^a^ H indicates healthy pig, D indicates diseased pig, and *P* indicates human patient.

^b^ The survival outcome of serotype 4 strains for zebrafish was compared with that of the highly pathogenic strain SC070731 using the Log-rank (Mantel-Cox) test. * indicates *p* < 0.05, ** indicates *p* < 0.01, *** indicates *p* < 0.001, **** indicates *p* < 0.0001, and “ns” indicates no significant difference.

/: unassigned.

The mouse infection experiment was performed further to validate the virulence of *S. suis* serotype 4 strains. Nine strains with ≥ 80% mortality to zebrafish and two strains with no mortality to zebrafish were selected for BALB/c mice infection. As shown in Table 3, the mortality rate of mice infected with eight strains with ≥ 80% mortality to zebrafish was also ≥ 80%. The survival curves of mice infected with strains ID36054, WUSS406, ND90, 2021WUSS075, 2018WUSS156, WUSS346, and 2018WUSS160 showed no significant difference compared to mice infected with the highly virulent control strain SC070731. Strain ND84, with 86.67% mortality to zebrafish, exhibited 60% mortality to mice. Two strains with no mortality to zebrafish also showed no mortality to mice. The results of mice and zebrafish infection experiments were consistent. In summary, 35.00% (7/20) of representative pig isolates of CC17, CC94, and CC839 were highly virulent in zebrafish and mice (mortality rate ≥ 80%), similar to the human isolate ID36054 (belonging to CC94) (Table 3). Thus, *S. suis* serotype 4 strains of CC17 (ST17), CC94 (ST94, ST2220), and CC839 (ST485) are potentially pathogenic.

**Table 3.** The results of the BALB/c mice infection experiment.

Strains	Mortality rate (%)^a^	CC	Deaths at different post-infection time points										Total deaths	Mortality rate (%)^b^	*P* value	Significance^c^
			1 d	2 d	3 d	4 d	5 d	6 d	7 d	8 d	9 d	10 d				
WUSS406	80.00	17	10	0	0	0	0	0	0	0	0	0	10	100.00	0.3173	ns
2018WUSS011	93.33	17	5	0	0	0	0	3	0	0	0	0	8	80.00	0.0111	*
ND84	86.67	17	6	0	0	0	0	0	0	0	0	0	6	60.00	0.0317	*
ND90	80.00	17	7	1	0	0	0	0	0	0	0	0	8	80.00	0.1512	ns
ID36054	93.33	94	9	1	0	0	0	0	0	0	0	0	10	100.00	>0.9999	ns
2021WUSS075	93.33	94	9	1	0	0	0	0	0	0	0	0	10	100.00	>0.9999	ns
2018WUSS156	86.67	94	6	2	0	0	0	0	0	0	0	0	8	80.00	0.0863	ns
WUSS346	93.33	839	10	0	0	0	0	0	0	0	0	0	10	100.00	0.3173	ns
2018WUSS160	100.00	839	9	0	0	0	0	0	0	0	0	0	9	90.00	0.5567	ns
2022WUSS018	0.00	/	0	0	0	0	0	0	0	0	0	0	0	0.00	<0.0001	****
2022WUSS056	0.00	/	0	0	0	0	0	0	0	0	0	0	0	0.00	<0.0001	****
SC070731	93.33	1	9	1	0	0	0	0	0	0	0	0	10	100.00	-	-
PBS	0.00		0	0	0	0	0	0	0	0	0	0	0	0.00	<0.0001	****

^a^ Mortality rate of each strain in zebrafish.

^b^ Mortality rate of each strain in BALB/c mice.

^c^ The survival outcome of serotype 4 strains for mice was compared with that of the highly pathogenic strain SC070731 using the Log-rank (Mantel-Cox) test. * indicates *p* < 0.05, ** indicates *p* < 0.01, *** indicates *p* < 0.001, **** indicates *p* < 0.0001, and “ns” indicates no significant difference.

/: unassigned.

### Cell cytotoxicity

**Figure 3.**
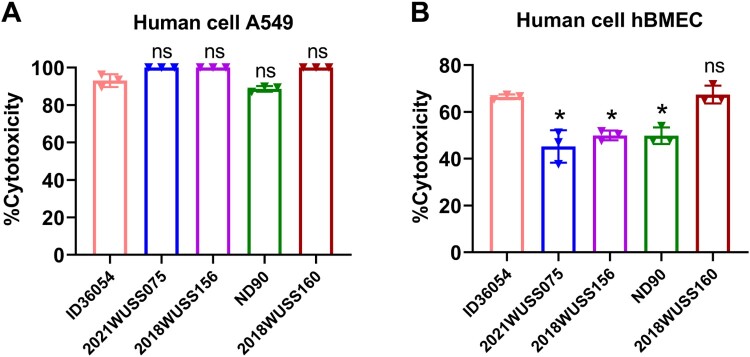
Human cell cytotoxicity assays of *S. suis* serotype 4 highly virulent strains. Cell cytotoxicity of *S. suis* strains on A549 (A) for 3 h and hBMEC (B) for 6 h. The percentage of cytotoxicity of *S. suis* serotype 4 strains was compared with that of the human reference strain ID36054 using an unpaired *t*-test. A summary of the *p*-values is provided, with an asterisk indicating a significant difference (*p* < 0.05) and “ns” denoting no significant difference.

To evaluate the cell cytotoxicity of highly virulent *S. suis* serotype 4 strains on human cells, the human isolate ID36054 and four pig isolates belonging to CC17, CC94, and CC839 were selected for cell cytotoxicity assays, and the mortality rate of these strains in both zebrafish and mice is ≥ 80%. As shown in Figure 3(A), the human strain ID36054 exhibited significant cytotoxicity to A549 at 3 h incubation with 93.04% cytotoxicity, and the pig isolates showed 88.67%−100.00% cytotoxicity. As shown in Figure 3(B), the human strain ID36054 exhibited significant cytotoxicity to hBMEC at 6 h incubation with 66.42% cytotoxicity, and the pig isolates showed 45.25%−67.41% cytotoxicity. These data indicate that the human and pig isolates of *S. suis* serotype 4 exhibit significant cytotoxicity to human cells.

### Distribution and dissemination vehicles of antibiotic resistance genes in *S. suis* serotype 4

**Figure 4.**
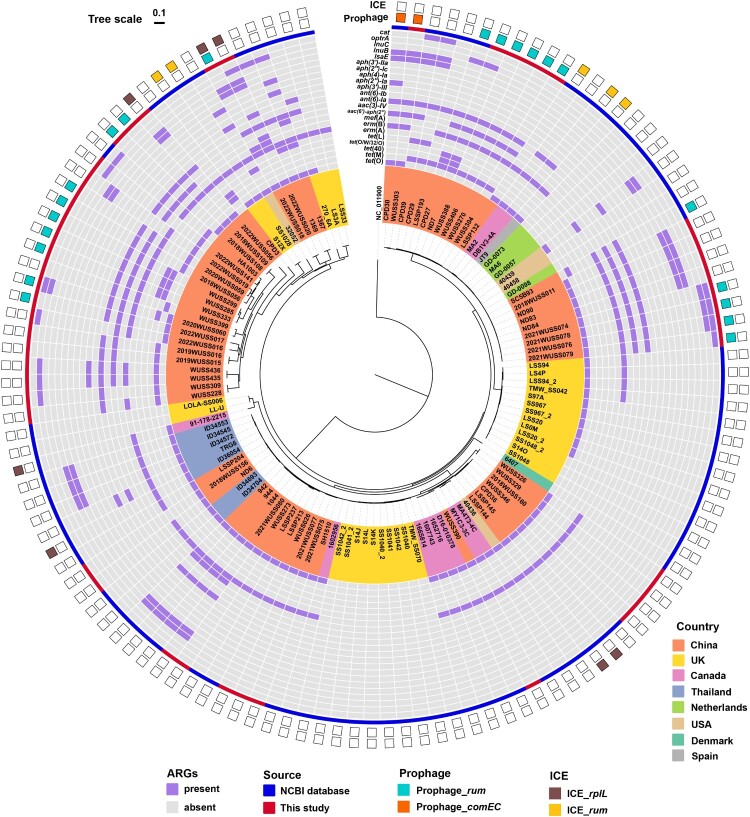
The distribution of antibiotic resistance genes (ARGs), prophages, and ICEs for *S. suis* serotype 4 genomes. Color-filled square boxes on the periphery indicate the presence of antibiotic resistance genes-associated prophages and ICEs, and unfilled boxes indicate their absence.

The distribution of antibiotic resistance genes in 126 *S. suis* serotype 4 genomes was investigated. We identified twenty-two distinct antibiotic resistance genes, which showed ≥ 95% identity in nucleotide sequence across over 90% coverage of the reference gene. They were classified into six categories: tetracyclines, macrolides-lincosamides-streptogramin B (MLS_B_), aminoglycosides, lincosamides, oxazolidinones, and chloramphenicol (Figure 4). The distribution of tetracycline resistance genes was found to be highest at a rate of 81.75% (103/126), corresponding to the phenotypes of resistance to doxycycline and tetracycline, with *tet*(O) being the predominant gene (77/126, 61.11%), followed by *tet*(M) (22/126, 17.46%), *tet*(40) (7/126, 5.56%), *tet*(O/W/32/O) (6/126, 4.76%), and *tet*(L) (3/126, 2.38%). Ninety-two genomes (73.02%) possess the MLS_B_ resistance gene *erm*(B), corresponding to the phenotypes of resistance to erythromycin, azithromycin, lincomycin, and clindamycin. Other lincosamides resistance genes *lsaE* (20/126, 15.87%), *lnuB* (20/126, 15.87%), and *lnuC* (2/126, 1.59%) were also found in 22 genomes (17.46%). Nine types of aminoglycoside resistant genes were found among 53 genomes (42.06%), consisting of *ant(6)-Ia* (51/126, 40.48%), *aac(6′)-aph(2″)* (36/126, 28.57%), *aph(3′)-III* (6/126, 4.76%), as well as one each of *ant(6)-Ib*, *aph(2″)-Ia*, *aph(2″)-Ic*, *aph(3′)-IIa*, *aph(4)-Ia*, and *aac(3)-IV*. The florfenicol and linezolid resistance gene *optrA* (12/126, 9.52%), chloramphenicol resistance gene *cat* (8/126, 6.35%), and efflux pump gene *mef*(A) (6/126, 4.76%) were also found in the *S. suis* serotype 4 genomes.

**Figure 5.**
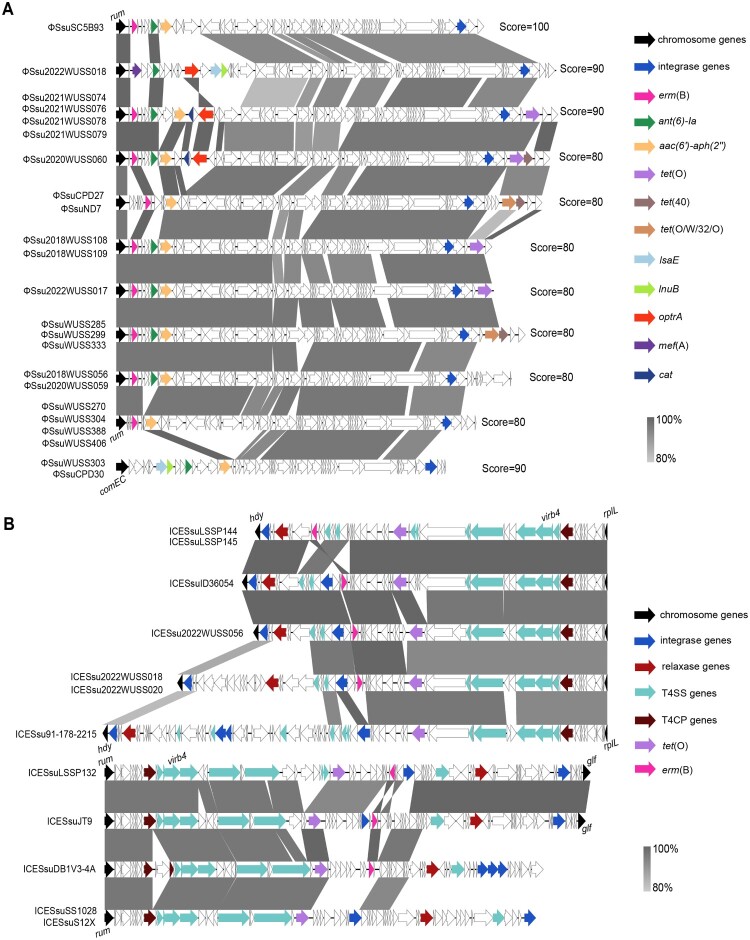
Genetic context of antibiotic resistance genes-associated prophages and ICEs in *S. suis* serotype 4 genomes. The direction of transcription for each gene is denoted by arrows, and distinct colours represent the various genes. (A) Prophages with scores of 100, 90, and 80 by PHASTER analysis. (B) ICE structure predicted by ICEfinder software. T4SS, type IV secretion system; T4CP, type IV coupling protein.

Our investigation successfully identified an intact prophage with a score of 100 in one genome. Moreover, questionable prophages with predicted scores of 90 and 80 were present in seven and fifteen genomes, respectively (Figure 4). Integration analysis revealed that these prophages were primarily inserted into the *rum* locus. However, prophages ΦSsuWUSS303 and ΦSsuCPD30 were inserted into the *comEC* locus instead. It is worth mentioning that the MLS_B_ resistance gene *erm*(B), aminoglycosides resistance genes *ant(6)-Ia* and *aac(6′)-aph(2*″*)*, tetracyclines resistance genes *tet*(O), *tet*(O/W/32/O), and *tet*(40), and other resistance genes *optrA*, *mef*(A), *lsaE*, *lnuB*, *lnuC*, and *cat* were detected within prophages (Figure 5(A)). In addition, nine different ICEs were distributed in 12 genomes, containing five ICEs inserted into the *rplL* locus and four ICEs inserted into the *rum* locus (Figure 4). These ICEs predominantly carried *erm*(B) and *tet*(O) genes (Figure 5(B)). We utilized online analysis platforms to detect conjugative plasmids carrying antibiotic resistance genes in the strains studied. The results indicate that no conjugative plasmids carrying antibiotic resistance genes were found. This discovery implies that prophages were the primary vehicle of antibiotic resistance genes and played a crucial role in disseminating MLS_B_, tetracyclines, aminoglycosides, and linezolid resistance genes. Additionally, the presence of ICEs further facilitated the dissemination of MLS_B_ and tetracycline resistance genes. We also observed that genomes harbouring prophages associated with antibiotic resistance genes were exclusively isolated from China, whereas those harbouring ICEs associated with antibiotic resistance genes were discovered mainly in other nations (Figure 4).

### Antimicrobial susceptibility profiles of *S. suis* serotype 4 strains

**Figure 6.**
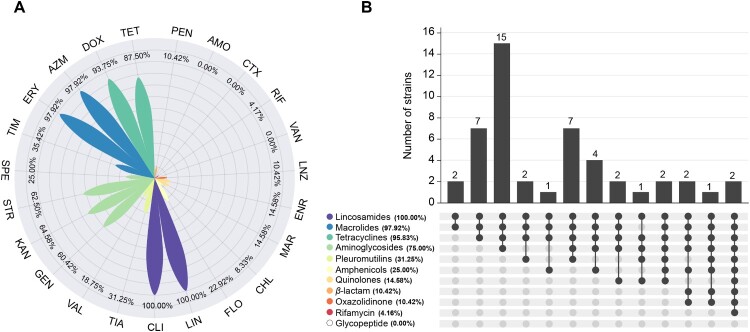
The antimicrobial susceptibility profiles in *S. suis* serotype 4 strains isolated from our labs. (A) The resistance rates of 48 strains to 23 antibiotics. PEN, Penicillin; AMO, Amoxicillin; CTX, Cefotaxime; RIF, Rifampin; VAN, Vancomycin; LNZ, Linezolid; ENR, Enrofloxacin; MAR, Marbofloxacin; CHL, Chloramphenicol; FLO, Florfenicol; LIN, Lincomycin; CLI, Clindamycin; TIA, Tiamulin; VAL, Valnemulin; GEN, Gentamicin; KAN, Kanamycin; STR, Streptomycin; SPE, Spectinomycin; TIM, Tilmicosin; ERY, Erythromycin; AZM, Azithromycin; DOX, Doxycycline; TET, Tetracycline. (B) The resistance rates of 48 strains to 11 categories of antibiotics.

We detected the antibiotic resistance phenotypes of 48 *S. suis* serotype 4 strains isolated from our labs using the broth microdilution method. The MIC values of 23 antimicrobials tested for 48 strains are listed in Table S2. As shown in Figure 6(A), all strains were resistant to lincomycin and clindamycin, and most strains were also resistant to erythromycin (97.92%), azithromycin (97.92%), doxycycline (93.75%), and tetracycline (87.50%). The presence of the genes *erm*(B) (89.58%), *lsaE* (20.83%), and *lnuB* (20.83%) conferred resistance to lincosamides and macrolides, and the presence of the genes *tet*(O) (60.42%), *tet*(M) (33.33%), and *tet*(O/W/32/O) (8.33%) conferred resistance to tetracyclines (Figure 4, Table S2). The resistance rate for kanamycin, streptomycin, gentamicin, tilmicosin, tiamulin, spectinomycin, florfenicol, and valnemulin was 64.58%, 62.50%, 60.42%, 35.42%, 31.25%, 25.00%, 22.92%, and 18.75%, respectively. The carriage of the genes *aac(6′)-aph(2″)* (64.58%) and *aph(3′)-III* (6.25%) led to high-level resistance to gentamicin and kanamycin, while the presence of the gene *ant(6)-Ia* (72.92%) resulted in high-level resistance to streptomycin (Figure 4, Table S2). All *S. suis* serotype 4 strains displayed susceptibility to amoxicillin, cefotaxime, and vancomycin. There was a low resistance rate to enrofloxacin (14.58%), marbofloxacin (14.58%), penicillin (10.42%), linezolid (10.42%), chloramphenicol (8.33%), and rifampin (4.17%). It is worth noting that 95.83% (46/48) of the strains were resistant to ≥3 classes of antimicrobial agents (Figure 6(B)) and were classified as multidrug-resistant. Most strains were resistant to four or five classes of antimicrobial agents.

## Discussion

Serotyping is vital for gaining insights into bacterial epidemiology, such as its prevalence across different geographic regions and transmission dynamics. Identifying specific serotypes or STs linked to outbreaks offer invaluable information for epidemiologists to comprehend disease spread and implement targeted control measures. Currently available vaccines against *S. suis* infection are mainly bacterins, which are supposed to confer serotype-specific protection. Thus, serotyping helps to develop serotype-specific vaccines based on epidemiological data.

In the recent study by Murray *et al*., ten pathogenic lineages of *S. suis* were identified, among which disease-associated serotypes 1, 1/2, 2, 3, 4, 5, 6, 7, 8, 9, and 14 were found to be prevalent [22]. However, different *S. suis* serotypes exhibit diverse population structure and pathogenicity characteristics. Therefore, for the pathogenicity of *S. suis*, it is important to not only focus on disease-associated serotypes but also ST [1,6]. *S. suis* serotype 2 is the most prevalent pathotype in both swine and humans worldwide [1]. The common pathogenic ST of *S. suis* serotype 2 are ST1, ST7, ST20, ST25, ST28, and ST104. Notably, ST1 strains belonging to MCG group 1 are globally distributed and demonstrate the highest pathogenicity in humans and pigs [6]. Besides this serotype, *S. suis* serotype 9 has emerged as the predominant serotype among diseased pigs in Western Europe; ST16 strains showed a zoonotic potential [1,47,48]. *S. suis* serotype 7, a non-negligible pathotype with the most predominant STs being ST29, ST373, and ST94, prevalent in Europe, China, and North America, respectively; ST373 strains were responsible for septicemia in humans and widely prevalent in China [14]. *S. suis* serotype 8, as one of the main pathogenic serotypes causing clinical diseases in pigs, was frequently isolated from clinical cases in Asia, North America, South America, and Europe [1,49,50]. Among the isolates of serotype 8 in China, the predominant STs included ST308, ST198, and ST1241, with the ST1241 strains showing significant pathogenicity in zebrafish and mice [40]. The main purpose of population structure analysis for different *S. suis* serotypes is to identify the high-pathogenic sub-lineages from the whole population and provide important information to precisely prevent the infection caused by the high-pathogenic sub-lineage strains.

Between 2002 and 2013, in China and South Korea, *S. suis* serotype 4 ranked as the third most predominant serotype isolated from infected pigs, accounting for 5.6% [1]. In Canada, there was an increasing trend in the proportion of *S. suis* serotype 4 among isolates from diseased pigs between 2009 and 2011, rising from 3.7% to 5.9% [18]. In Germany, *S. suis* serotype 4 accounted for 10% and 10.3% of *S. suis* isolates identified between 1996–2004 and 2015–2016, respectively [20]. In this study, the results of animal infection experiments showed that among the representative strains of CC17 (ST17), CC94 (ST94, ST2220), and CC839 (ST485), 66.67% (14/21) of the strains exhibited a mortality rate exceeding 50% in zebrafish (Table 2). Therefore, *S. suis* serotype 4 is a non-negligible pathotype.

In a recent study, Hatrongjit *et al*. analyzed the genome of seven serotype 4 strains belonging to CC94 and group 3 [26]; they performed cytotoxicity assays using human cell lines and demonstrated that CC94 serotype 4 strains are potentially virulent [26]. However, the virulence of those strains was not validated in animal models, and only seven strains were analyzed in their study. In the present study, we investigated the population structure and pathogenicity of the *S. suis* serotype 4 population based on 126 isolates from eight different countries. Within the *S. suis* serotype 4 population, CC17 and CC94, which belong to MCG group 1 and MCG group 3, respectively, exhibited the highest proportions. Chen *et al*. reported seven MCG groups among *S. suis* population; MCG group 1 included all the highly virulent isolates of ST1, the epidemic isolates of ST7, and all isolates from human infections and outbreaks [28]. Notably, *S. suis* serotype 4 strain ID36054 causing human infection belongs to CC94. In this study, the results of animal infection experiments showed that 35.00% (7/20) of pig isolates from CC17, CC94, and CC839 (also belonging to MCG group 3) were highly virulent in zebrafish and mice, similar to the human isolate ID36054. We found a correlation between the virulence phenotype and the distribution of putative virulence-related genes. These highly virulent strains harbour numerous crucial genes associated with virulence, particularly including *mrp*, *sly*, *IdeSsuis*, *igdE*, *sbp2*, *hp0197*, *hp0272*, *rgg*, and *tran* which have been identified as potential zoonotic virulence factors in a recent study [51]. In addition, we found that the genotype of *S. suis* classical virulence markers in highly virulent strains was *ef*+/*mrp*^NA2^/*sly*+ or *ef*-/*mrp*^NA1^/*sly*+. Notably, the genotype of *ef*+/*mrp*^NA2^/*sly*+ was prevalent in human infection strains [52,53]. In contrast, all strains from MCG groups 7-2 and 7-3 were lowly virulent in zebrafish. Compared to the highly virulent strains, they lack several crucial virulence genes, including *ef*, *mrp*, *sly*, *ofs*, *sao*, *dltA*, *IdeSsuis*, *igdE*, *revS*, *rgg*, and *tran*. Furthermore, cell cytotoxicity assays confirmed that the human and pig isolates of *S. suis* serotype 4 exhibit significant cytotoxicity to human cells A549 and hBMEC. Thus, *S. suis* serotype 4 strains of CC17, CC94, and CC839 exhibit significant threat to humans and pigs and should be monitored.

Previous studies have shown *S. suis* to be a reservoir of clinically significant antibiotic resistance genes for major streptococcal pathogens [35,54,55]. In this study, we observed that 95.83% *S. suis* serotype 4 strains isolated from our labs were multidrug-resistant. The presence of *erm*(B), *tet* genes, *ant(6)-Ia*, *aac(6′)-aph(2″)*, and *optrA* were the main reasons for the prevalent resistance phenotypes to macrolides, lincosamides, tetracyclines, aminoglycosides, and linezolid in serotype 4 strains. Prophages were identified as their primary vehicle, and ICEs further facilitated the dissemination of *erm*(B) and *tet*(O). In *Streptococcus* species, the dissemination of antibiotic resistance genes is primarily facilitated through the horizontal gene transfer of ICEs and prophages [35,56–60]. However, major vehicles may differ for *S. suis* serotypes. For example, prophages were the primary vehicle of antibiotic resistance genes in serotype 31 strains [41]. In serotype 7 and 8 strains, antibiotic resistance genes were mainly disseminated by integrative and mobilizable elements (IMEs) [40]. We found that *S. suis* serotype 4 genomes containing prophages associated with antibiotic resistance genes were mainly isolated from China, while those containing ICEs associated with antibiotic resistance genes were mainly discovered in other countries, suggests that the predominant vehicles for the spread of antibiotic resistance genes may vary by nation or region.

In conclusion, *S. suis* serotype 4 exhibited a distinct population structure. 61.90% of strains (78/126) were clustered into MCG groups 1 and 3, with potential pathogenicity in humans and pigs, particularly strains belonging to CC17, CC94, and CC839. *S. suis* serotype 4 strains showed multidrug-resistant, with prophages crucial in disseminating antibiotic resistance genes. Our study expands the understanding of the *S. suis* serotype 4 population and provides valuable information for the surveillance and prevention of this serotype.

## Supplementary Material

Supplemental Material
