# Supplementary material for: Streptococcus suis serotype 4: a population with the potential pathogenicity in humans and pigs
Source: Emerg Microbes Infect. 2024 May 4;13(1):2352435. doi: 10.1080/22221751.2024.2352435 (PMC11097711; doi:10.1080/22221751.2024.2352435)
Supplement: Supplemental Material [file TEMI_A_2352435_SM3774.docx]

Supplemental Table 1. The 35 putative virulence-associated genes analyzed in this study.

| **Genes** | **Annotation/ Function** | | **Critical role**  **in virulence ^a^** |
| --- | --- | --- | --- |
| **Adhesins and cell surface factors** | **Annotation** |  | |
| *atl_2* | Autolysin | | Yes |
| *ef* | Extracellular factor (EF) | | No |
| *enolase* | Enolase | | NT |
| *fbps* | Fibronectin-fibrinogen binding protein (FBPS) | | Yes |
| *hp0197* | HP0197 | | Yes |
| *hp0272* | HP0272 | | Yes |
| *HtpsC* | Type II histidine triad protein (HtpsC) | | Yes |
| *mrp* | Muraminidase released protein (MRP) | | No |
| *ofs* | Serum opacity factor (OFS) | | Yes |
| *oppA* | Oligopeptide-binding protein precursor (OppA) | | Yes |
| *sao* | Surface antigen one (Sao) | | No |
| *sbp2* | Putative pilin subunit (Sbp2) | | Yes |
| *srtA* | Sortase A | | Yes |
| *ss05_1311* | Fibronectin-binding protein (Ssa) | | No |
| **Toxins and inflammation** | **Annotation** |  | |
| *abpb* | Amylase-binding protein B (Abpb) | | Yes |
| *clpP* | Degradative enzyme ClpP | | Yes |
| *clpX* | Degradative enzyme ClpX | | Yes |
| *Hp1717* | HP1717 | | Yes |
| *sly* | Suilysin | | No |
| *SspA* | Surface-associated subtilisin-like protease (SspA) | | Yes |
| **Immunoevasion** | **Annotation** |  | |
| *dltA* | DltA | | Yes |
| *DPP IV* | Dipeptidyl peptidase IV (DPP IV) | | Yes |
| *IdeSsuis* | IdeSsuis | | Yes |
| *igdE* | IgdE | | Yes |
| *pgdA* | PgdA | | Yes |
| *SntA* | SntA | | NT |
| *SsnA* | SsnA | | NT |
| **Regulatory factors** | **Function** | |  |
| *codY* | DNA-binding repressor | | Yes |
| *gidA* | Glucose-inhibited division protein (GidA) | | Yes |
| *luxS* | Quorum sensing component | | Yes |
| *revS* | Orphan response regulator | | Yes |
| *rgg* | Transcriptional regulator | | Yes |
| *stk* | Signal transduction regulation | | Yes |
| *stp1* | Serine threonine phosphatase (Stp 1) | | Yes |
| *tran* | Transcriptional regulator | | Yes |

^a^ The critical role was evaluated by knockout mutants in animal models.

NT: The virulence of mutant was not tested in animal models.

Supplemental Table 2. Antimicrobial susceptibility profiles of available *S. suis* serotype 4 strains.

| **Strains** | **tested antimicrobial agents and breakpoints for resistant (µg/mL)** | | | | | | | | | | | |
| --- | --- | --- | --- | --- | --- | --- | --- | --- | --- | --- | --- | --- |
|  | **penicillin** | **amoxicillin** | **cefotaxime** | **rifampin** | **vancomycin** | **linezolid** | **enrofloxacin** | **marbofloxacin** | **chloramphenicol** | **florfenicol** | **lincomycin** | **clindamycin** |
|  | ≥ 1 | ˃ 2 | ≥ 8 | ≥ 4 | ˃ 1 | ˃ 2 | ≥ 2 | ≥ 2 | ≥ 16 | ≥ 8 | ≥ 1 | ≥ 1 |
|  | **MIC value of *S. suis* serotype 4 strains (µg/mL)** | | | | | | | | | | | |
| WUSS026 | ≤ 0.5 | ≤ 0.5 | ≤ 0.5 | ≤ 0.5 | ≤ 0.5 | ≤ 0.5 | ≤ 0.5 | ≤ 0.5 | 4 | 2 | > 256 | > 256 |
| WUSS228 | ≤ 0.5 | ≤ 0.5 | ≤ 0.5 | ≤ 0.5 | ≤ 0.5 | 1 | 8 | 16 | 2 | 2 | 16 | > 256 |
| WUSS270 | ≤ 0.5 | ≤ 0.5 | ≤ 0.5 | ≤ 0.5 | ≤ 0.5 | ≤ 0.5 | ≤ 0.5 | ≤ 0.5 | 2 | 2 | > 256 | > 256 |
| WUSS273 | ≤ 0.5 | ≤ 0.5 | ≤ 0.5 | ≤ 0.5 | ≤ 0.5 | ≤ 0.5 | ≤ 0.5 | ≤ 0.5 | 2 | 2 | > 256 | > 256 |
| WUSS285 | ≤ 0.5 | ≤ 0.5 | ≤ 0.5 | ≤ 0.5 | ≤ 0.5 | ≤ 0.5 | 16 | 16 | 4 | 4 | > 256 | > 256 |
| WUSS299 | ≤ 0.5 | ≤ 0.5 | ≤ 0.5 | ≤ 0.5 | ≤ 0.5 | 1 | 16 | 16 | 4 | 4 | > 256 | > 256 |
| WUSS303 | ≤ 0.5 | ≤ 0.5 | ≤ 0.5 | ≤ 0.5 | ≤ 0.5 | 1 | ≤ 0.5 | ≤ 0.5 | 2 | 2 | > 256 | > 256 |
| WUSS304 | ≤ 0.5 | ≤ 0.5 | ≤ 0.5 | ≤ 0.5 | ≤ 0.5 | ≤ 0.5 | ≤ 0.5 | 1 | 4 | 2 | > 256 | > 256 |
| WUSS309 | ≤ 0.5 | ≤ 0.5 | ≤ 0.5 | ≤ 0.5 | ≤ 0.5 | 2 | 8 | 16 | 8 | 32 | > 256 | > 256 |
| WUSS326 | ≤ 0.5 | ≤ 0.5 | ≤ 0.5 | ≤ 0.5 | ≤ 0.5 | ≤ 0.5 | ≤ 0.5 | ≤ 0.5 | ≤ 0.5 | 1 | > 256 | > 256 |
| WUSS329 | ≤ 0.5 | ≤ 0.5 | ≤ 0.5 | ≤ 0.5 | ≤ 0.5 | ≤ 0.5 | ≤ 0.5 | ≤ 0.5 | ≤ 0.5 | 1 | 256 | > 256 |
| WUSS333 | ≤ 0.5 | ≤ 0.5 | ≤ 0.5 | ≤ 0.5 | ≤ 0.5 | ≤ 0.5 | ≤ 0.5 | ≤ 0.5 | 4 | 2 | > 256 | > 256 |
| WUSS346 | ≤ 0.5 | ≤ 0.5 | ≤ 0.5 | ≤ 0.5 | ≤ 0.5 | ≤ 0.5 | ≤ 0.5 | ≤ 0.5 | 2 | 2 | > 256 | > 256 |
| WUSS388 | ≤ 0.5 | ≤ 0.5 | ≤ 0.5 | ≤ 0.5 | ≤ 0.5 | ≤ 0.5 | ≤ 0.5 | ≤ 0.5 | 2 | 2 | > 256 | > 256 |
| WUSS390 | ≤ 0.5 | ≤ 0.5 | ≤ 0.5 | ≤ 0.5 | ≤ 0.5 | ≤ 0.5 | ≤ 0.5 | ≤ 0.5 | 2 | 1 | > 256 | > 256 |
| WUSS399 | ≤ 0.5 | ≤ 0.5 | ≤ 0.5 | ≤ 0.5 | ≤ 0.5 | ≤ 0.5 | ≤ 0.5 | ≤ 0.5 | 4 | 2 | > 256 | > 256 |
| WUSS406 | ≤ 0.5 | ≤ 0.5 | ≤ 0.5 | ≤ 0.5 | ≤ 0.5 | ≤ 0.5 | ≤ 0.5 | ≤ 0.5 | 2 | 2 | > 256 | > 256 |
| WUSS435 | 2 | ≤ 0.5 | ≤ 0.5 | 32 | ≤ 0.5 | 4 | 32 | 32 | 32 | 64 | > 256 | > 256 |
| WUSS436 | 2 | ≤ 0.5 | ≤ 0.5 | 32 | ≤ 0.5 | 4 | 32 | 32 | 32 | 64 | > 256 | > 256 |
| 2018WUSS011 | ≤ 0.5 | ≤ 0.5 | ≤ 0.5 | ≤ 0.5 | ≤ 0.5 | ≤ 0.5 | ≤ 0.5 | ≤ 0.5 | 2 | 2 | > 256 | > 256 |
| 2018WUSS056 | ≤ 0.5 | ≤ 0.5 | ≤ 0.5 | ≤ 0.5 | ≤ 0.5 | ≤ 0.5 | ≤ 0.5 | ≤ 0.5 | 2 | 2 | > 256 | > 256 |
| 2018WUSS108 | ≤ 0.5 | ≤ 0.5 | ≤ 0.5 | ≤ 0.5 | ≤ 0.5 | ≤ 0.5 | ≤ 0.5 | ≤ 0.5 | 2 | 2 | > 256 | > 256 |
| 2018WUSS109 | ≤ 0.5 | ≤ 0.5 | ≤ 0.5 | ≤ 0.5 | ≤ 0.5 | 1 | ≤ 0.5 | ≤ 0.5 | 4 | 2 | > 256 | > 256 |
| 2018WUSS156 | ≤ 0.5 | ≤ 0.5 | ≤ 0.5 | ≤ 0.5 | ≤ 0.5 | ≤ 0.5 | ≤ 0.5 | ≤ 0.5 | 2 | 2 | > 256 | > 256 |
| 2018WUSS160 | ≤ 0.5 | ≤ 0.5 | ≤ 0.5 | ≤ 0.5 | ≤ 0.5 | ≤ 0.5 | ≤ 0.5 | ≤ 0.5 | 2 | 2 | > 256 | > 256 |
| 2019WUSS015 | ≤ 0.5 | ≤ 0.5 | ≤ 0.5 | ≤ 0.5 | ≤ 0.5 | ≤ 0.5 | ≤ 0.5 | ≤ 0.5 | 2 | 2 | > 256 | > 256 |
| 2019WUSS016 | ≤ 0.5 | ≤ 0.5 | ≤ 0.5 | ≤ 0.5 | ≤ 0.5 | ≤ 0.5 | ≤ 0.5 | ≤ 0.5 | 2 | 2 | > 256 | > 256 |
| 2020WUSS059 | ≤ 0.5 | ≤ 0.5 | ≤ 0.5 | ≤ 0.5 | ≤ 0.5 | ≤ 0.5 | ≤ 0.5 | 1 | 2 | 2 | 256 | > 256 |
| 2020WUSS060 | ≤ 0.5 | ≤ 0.5 | ≤ 0.5 | ≤ 0.5 | ≤ 0.5 | 1 | 16 | 32 | 8 | 32 | > 256 | > 256 |
| 2021WUSS074 | ≤ 0.5 | ≤ 0.5 | ≤ 0.5 | ≤ 0.5 | ≤ 0.5 | 2 | ≤ 0.5 | ≤ 0.5 | 2 | 8 | > 256 | > 256 |
| 2021WUSS075 | ≤ 0.5 | ≤ 0.5 | ≤ 0.5 | ≤ 0.5 | ≤ 0.5 | 1 | ≤ 0.5 | 1 | 8 | 8 | > 256 | > 256 |
| 2021WUSS076 | ≤ 0.5 | ≤ 0.5 | ≤ 0.5 | ≤ 0.5 | ≤ 0.5 | 2 | ≤ 0.5 | ≤ 0.5 | 4 | 8 | > 256 | > 256 |
| 2021WUSS077 | ≤ 0.5 | ≤ 0.5 | ≤ 0.5 | ≤ 0.5 | ≤ 0.5 | 1 | ≤ 0.5 | ≤ 0.5 | 4 | 2 | > 256 | > 256 |
| 2021WUSS078 | ≤ 0.5 | ≤ 0.5 | ≤ 0.5 | ≤ 0.5 | ≤ 0.5 | 1 | ≤ 0.5 | ≤ 0.5 | 8 | 8 | > 256 | > 256 |
| 2021WUSS079 | ≤ 0.5 | ≤ 0.5 | ≤ 0.5 | ≤ 0.5 | ≤ 0.5 | 1 | ≤ 0.5 | 1 | 4 | 1 | > 256 | > 256 |
| 2021WUSS080 | ≤ 0.5 | ≤ 0.5 | ≤ 0.5 | ≤ 0.5 | ≤ 0.5 | 1 | ≤ 0.5 | ≤ 0.5 | 2 | ≤ 0.5 | > 256 | > 256 |
| 2022WUSS016 | ≤ 0.5 | ≤ 0.5 | ≤ 0.5 | ≤ 0.5 | ≤ 0.5 | ≤ 0.5 | ≤ 0.5 | ≤ 0.5 | 16 | 2 | 256 | > 256 |
| 2022WUSS017 | ≤ 0.5 | ≤ 0.5 | ≤ 0.5 | ≤ 0.5 | ≤ 0.5 | 1 | ≤ 0.5 | 1 | 4 | 2 | 256 | > 256 |
| 2022WUSS018 | 8 | ≤ 0.5 | ≤ 0.5 | ≤ 0.5 | ≤ 0.5 | 4 | ≤ 0.5 | ≤ 0.5 | 8 | 32 | 128 | > 256 |
| 2022WUSS019 | ≤ 0.5 | ≤ 0.5 | ≤ 0.5 | ≤ 0.5 | ≤ 0.5 | ≤ 0.5 | ≤ 0.5 | ≤ 0.5 | 2 | 1 | 256 | > 256 |
| 2022WUSS020 | 1 | ≤ 0.5 | ≤ 0.5 | ≤ 0.5 | ≤ 0.5 | 4 | ≤ 0.5 | ≤ 0.5 | 8 | 16 | 128 | > 256 |
| 2022WUSS056 | ≤ 0.5 | ≤ 0.5 | ≤ 0.5 | ≤ 0.5 | ≤ 0.5 | ≤ 0.5 | ≤ 0.5 | ≤ 0.5 | 2 | 1 | > 256 | > 256 |
| 2022WUSS141 | 8 | ≤ 0.5 | ≤ 0.5 | ≤ 0.5 | ≤ 0.5 | 4 | ≤ 0.5 | ≤ 0.5 | 16 | 16 | > 256 | > 256 |
| ND6 | ≤ 0.5 | ≤ 0.5 | ≤ 0.5 | ≤ 0.5 | ≤ 0.5 | 1 | ≤ 0.5 | ≤ 0.5 | 4 | 2 | > 256 | > 256 |
| ND7 | ≤ 0.5 | ≤ 0.5 | ≤ 0.5 | ≤ 0.5 | ≤ 0.5 | 1 | ≤ 0.5 | ≤ 0.5 | 4 | 4 | > 256 | > 256 |
| ND83 | ≤ 0.5 | ≤ 0.5 | ≤ 0.5 | ≤ 0.5 | ≤ 0.5 | ≤ 0.5 | ≤ 0.5 | ≤ 0.5 | 4 | 2 | > 256 | > 256 |
| ND84 | ≤ 0.5 | ≤ 0.5 | ≤ 0.5 | ≤ 0.5 | ≤ 0.5 | ≤ 0.5 | ≤ 0.5 | ≤ 0.5 | 2 | 2 | > 256 | > 256 |
| ND90 | ≤ 0.5 | ≤ 0.5 | ≤ 0.5 | ≤ 0.5 | ≤ 0.5 | ≤ 0.5 | ≤ 0.5 | ≤ 0.5 | 4 | 2 | > 256 | > 256 |

Supplemental Table 2 continued

| **Strains** | **tested antimicrobial agents and breakpoints for resistant (µg/mL)** | | | | | | | | | | | |
| --- | --- | --- | --- | --- | --- | --- | --- | --- | --- | --- | --- | --- |
|  | **tiamulin** | **valnemulin** | **gentamicin** | **kanamycin** | **streptomycin** | **spectinomycin** | **tilmicosin** | **erythromycin** | **azithromycin** | **doxycycline** | **tetracycline** |  |
|  | ≥ 32 | ≥ 32 | ≥ 16 | ˃ 256 | ˃ 256 | ≥ 128 | ≥ 32 | ≥ 1 | ≥ 2 | ˃ 1 | ˃ 8 |  |
|  | **MIC value of *S. suis* serotype 4 strains (µg/mL)** | | | | | | | | | | | |
| WUSS026 | 1 | ≤ 0.5 | 2 | 16 | 16 | 32 | ≤ 0.5 | 64 | > 256 | 2 | 16 |  |
| WUSS228 | 64 | 64 | 16 | > 256 | > 256 | > 256 | 2 | ≤ 0.5 | ≤ 0.5 | 8 | 64 |  |
| WUSS270 | ≤ 0.5 | ≤ 0.5 | > 256 | > 256 | > 256 | 64 | ≤ 0.5 | 256 | 256 | 2 | 16 |  |
| WUSS273 | 32 | ≤ 0.5 | 2 | 16 | 16 | 32 | ≤ 0.5 | 256 | > 256 | 4 | 16 |  |
| WUSS285 | 16 | 8 | > 256 | > 256 | > 256 | 32 | ≤ 0.5 | > 256 | > 256 | 4 | 64 |  |
| WUSS299 | 16 | 16 | 256 | > 256 | > 256 | 32 | ≤ 0.5 | > 256 | > 256 | 4 | 32 |  |
| WUSS303 | 8 | 2 | > 256 | > 256 | > 256 | 32 | ≤ 0.5 | 256 | 256 | 8 | 32 |  |
| WUSS304 | ≤ 0.5 | ≤ 0.5 | > 256 | > 256 | > 256 | 64 | ≤ 0.5 | 256 | > 256 | 4 | 16 |  |
| WUSS309 | 32 | 32 | 16 | > 256 | > 256 | > 256 | 2 | > 256 | > 256 | 8 | 32 |  |
| WUSS326 | ≤ 0.5 | ≤ 0.5 | 2 | 8 | 8 | 16 | ≤ 0.5 | 1 | 64 | ≤ 0.5 | 8 |  |
| WUSS329 | ≤ 0.5 | ≤ 0.5 | 2 | 16 | 8 | 16 | ≤ 0.5 | 1 | 8 | ≤ 0.5 | 8 |  |
| WUSS333 | 8 | 16 | 128 | > 256 | 256 | 16 | ≤ 0.5 | > 256 | > 256 | 4 | 32 |  |
| WUSS346 | ≤ 0.5 | ≤ 0.5 | ≤ 0.5 | 8 | 4 | 32 | ≤ 0.5 | 32 | 256 | 4 | 32 |  |
| WUSS388 | ≤ 0.5 | ≤ 0.5 | > 256 | > 256 | 256 | 32 | ≤ 0.5 | 256 | 256 | 2 | 16 |  |
| WUSS390 | 1 | ≤ 0.5 | 2 | 8 | 32 | 32 | ≤ 0.5 | 8 | 128 | 4 | 16 |  |
| WUSS399 | 16 | 32 | 128 | > 256 | > 256 | 32 | ≤ 0.5 | 256 | > 256 | 4 | 32 |  |
| WUSS406 | ≤ 0.5 | ≤ 0.5 | > 256 | > 256 | 256 | 32 | ≤ 0.5 | > 256 | > 256 | 4 | 32 |  |
| WUSS435 | 128 | 128 | > 256 | > 256 | > 256 | > 256 | 32 | > 256 | > 256 | 8 | 32 |  |
| WUSS436 | 128 | 128 | > 256 | > 256 | > 256 | > 256 | 32 | > 256 | > 256 | 8 | 32 |  |
| 2018WUSS011 | 32 | 2 | > 256 | > 256 | > 256 | > 256 | ≤ 0.5 | 256 | 256 | 4 | 32 |  |
| 2018WUSS056 | 8 | 16 | 256 | > 256 | > 256 | 32 | ≤ 0.5 | > 256 | > 256 | 4 | 32 |  |
| 2018WUSS108 | 8 | 8 | > 256 | > 256 | > 256 | 64 | 1 | > 256 | > 256 | 4 | 32 |  |
| 2018WUSS109 | 8 | 8 | 256 | > 256 | > 256 | 64 | ≤ 0.5 | > 256 | > 256 | 4 | 32 |  |
| 2018WUSS156 | 1 | ≤ 0.5 | 2 | 4 | 8 | 32 | ≤ 0.5 | > 256 | > 256 | 4 | 32 |  |
| 2018WUSS160 | 1 | ≤ 0.5 | 2 | 8 | 8 | 32 | 2 | 128 | > 256 | 8 | 32 |  |
| 2019WUSS015 | 32 | 32 | 256 | > 256 | > 256 | 32 | ≤ 0.5 | 32 | 256 | 4 | 32 |  |
| 2019WUSS016 | 32 | 32 | 256 | > 256 | > 256 | 32 | ≤ 0.5 | 128 | 256 | 4 | 32 |  |
| 2020WUSS059 | 4 | 4 | 256 | > 256 | > 256 | 16 | > 256 | 256 | 256 | 32 | 64 |  |
| 2020WUSS060 | 64 | 32 | > 256 | > 256 | > 256 | 16 | > 256 | 256 | 128 | 32 | 128 |  |
| 2021WUSS074 | ≤ 0.5 | ≤ 0.5 | 256 | > 256 | > 256 | 64 | > 256 | 4 | 16 | 4 | 32 |  |
| 2021WUSS075 | ≤ 0.5 | ≤ 0.5 | 2 | 16 | 4 | 32 | > 256 | 128 | 256 | 16 | 64 |  |
| 2021WUSS076 | ≤ 0.5 | ≤ 0.5 | > 256 | > 256 | > 256 | 32 | > 256 | 8 | 64 | 16 | 64 |  |
| 2021WUSS077 | 1 | ≤ 0.5 | 2 | 16 | 8 | 16 | > 256 | 256 | 256 | 4 | 8 |  |
| 2021WUSS078 | ≤ 0.5 | ≤ 0.5 | > 256 | > 256 | > 256 | 16 | > 256 | 64 | 64 | 2 | 1 |  |
| 2021WUSS079 | ≤ 0.5 | ≤ 0.5 | > 256 | > 256 | > 256 | 32 | > 256 | 128 | 128 | 4 | 1 |  |
| 2021WUSS080 | 32 | 1 | 2 | 8 | > 256 | > 256 | > 256 | > 256 | > 256 | 2 | 4 |  |
| 2022WUSS016 | 4 | 4 | 16 | 128 | 8 | 16 | > 256 | 256 | 256 | 16 | 64 |  |
| 2022WUSS017 | ≤ 0.5 | ≤ 0.5 | 256 | > 256 | > 256 | 16 | > 256 | 256 | 256 | 16 | 64 |  |
| 2022WUSS018 | 16 | 16 | 8 | 64 | > 256 | > 256 | > 256 | 2 | 4 | 16 | 64 |  |
| 2022WUSS019 | 64 | 16 | 4 | 64 | 16 | 16 | > 256 | > 256 | > 256 | 8 | 64 |  |
| 2022WUSS020 | 16 | 16 | > 256 | > 256 | > 256 | > 256 | > 256 | 2 | 2 | 16 | 64 |  |
| 2022WUSS056 | 16 | 1 | 4 | 32 | 256 | > 256 | > 256 | 256 | 256 | 16 | 64 |  |
| 2022WUSS141 | 64 | 64 | 2 | 128 | > 256 | 32 | ≤ 0.5 | > 256 | > 256 | 32 | 256 |  |
| ND6 | 2 | ≤ 0.5 | 1 | 8 | 8 | 16 | ≤ 0.5 | > 256 | > 256 | 4 | 16 |  |
| ND7 | ≤ 0.5 | ≤ 0.5 | 256 | > 256 | 256 | 8 | ≤ 0.5 | > 256 | > 256 | 8 | 32 |  |
| ND83 | 64 | 4 | ≤ 0.5 | > 256 | > 256 | > 256 | ≤ 0.5 | > 256 | > 256 | 8 | 16 |  |
| ND84 | 32 | 4 | ≤ 0.5 | > 256 | > 256 | > 256 | ≤ 0.5 | > 256 | > 256 | 4 | 16 |  |
| ND90 | 32 | 2 | ≤ 0.5 | > 256 | > 256 | > 256 | ≤ 0.5 | > 256 | > 256 | 4 | 16 |  |
